# 5-Bromo-2-(3-fluoro­phen­yl)-3-methyl­sulfinyl-1-benzofuran

**DOI:** 10.1107/S1600536812016789

**Published:** 2012-04-21

**Authors:** Hong Dae Choi, Pil Ja Seo, Uk Lee

**Affiliations:** a Department of Chemistry, Dongeui University, San 24 Kaya-dong Busanjin-gu, Busan 614-714, Republic of Korea; b Department of Chemistry, Pukyong National University, 599-1 Daeyeon 3-dong, Nam-gu, Busan 608-737, Republic of Korea

## Abstract

In the title compound, C_15_H_10_BrFO_2_S, the 3-fluoro­phenyl ring makes a dihedral angle of 30.77 (6)° with the mean plane [mean deviation = 0.014 (1) Å] of the benzofuran ring system. In the crystal, mol­ecules are linked by pairs of weak C—H⋯O hydrogen bonds into inversion dimers. A Br⋯O contact [3.214 (1) Å] is also observed.

## Related literature

For background information and the crystal structures of related compounds, see: Choi *et al.* (2007 ▶, 2010 ▶). For a review of halogen bonding, see: Politzer *et al.* (2007 ▶).
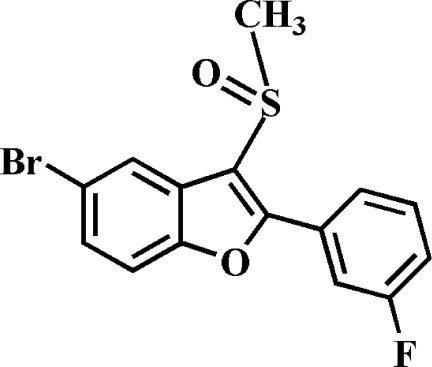


## Experimental

### 

#### Crystal data

C_15_H_10_BrFO_2_S
*M* *_r_* = 353.20
Triclinic, 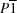

*a* = 8.0494 (1) Å
*b* = 8.5317 (1) Å
*c* = 10.7110 (2) Å
α = 87.624 (1)°
β = 81.378 (1)°
γ = 66.709 (1)°
*V* = 667.87 (2) Å^3^
*Z* = 2
Mo *K*α radiation
μ = 3.24 mm^−1^
*T* = 173 K
0.36 × 0.29 × 0.25 mm

#### Data collection

Bruker SMART APEXII CCD diffractometer
Absorption correction: multi-scan (*SADABS*; Bruker, 2009 ▶) *T*_min_ = 0.391, *T*_max_ = 0.501
11667 measured reflections
3059 independent reflections
2835 reflections with *I* > 2σ(*I*)
*R*_int_ = 0.038

#### Refinement

*R*[*F* ^2^ > 2σ(*F* ^2^)] = 0.025
*wR*(*F* ^2^) = 0.064
*S* = 1.07
3059 reflections
182 parameters
H-atom parameters constrained
Δρ_max_ = 0.36 e Å^−3^
Δρ_min_ = −0.46 e Å^−3^

### 

Data collection: *APEX2* (Bruker, 2009 ▶); cell refinement: *SAINT* (Bruker, 2009 ▶); data reduction: *SAINT*; program(s) used to solve structure: *SHELXS97* (Sheldrick, 2008 ▶); program(s) used to refine structure: *SHELXL97* (Sheldrick, 2008 ▶); molecular graphics: *ORTEP-3* (Farrugia, 1997 ▶) and *DIAMOND* (Brandenburg, 1998 ▶); software used to prepare material for publication: *SHELXL97*.

## Supplementary Material

Crystal structure: contains datablock(s) global, I. DOI: 10.1107/S1600536812016789/is5120sup1.cif

Structure factors: contains datablock(s) I. DOI: 10.1107/S1600536812016789/is5120Isup2.hkl

Supplementary material file. DOI: 10.1107/S1600536812016789/is5120Isup3.cml

Additional supplementary materials:  crystallographic information; 3D view; checkCIF report

## Figures and Tables

**Table 1:** Hydrogen-bond geometry (Å, °)

*D*—H⋯*A*	*D*—H	H⋯*A*	*D*⋯*A*	*D*—H⋯*A*
C13—H13⋯O2^i^	0.95	2.55	3.356 (2)	142

Symmetry code: (i) 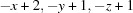.

**Fig. 1.**
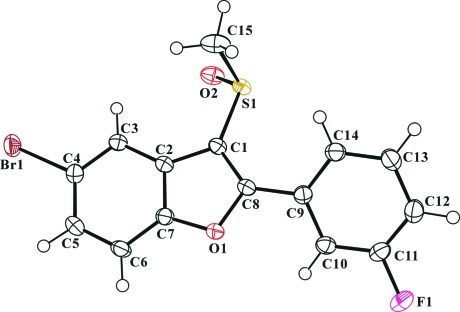
The molecular structure of the title compound with the atom numbering scheme. Displacement ellipsoids are drawn at the 50% probability level. H atoms are presented as small spheres of arbitrary radius.

**Fig. 2.**
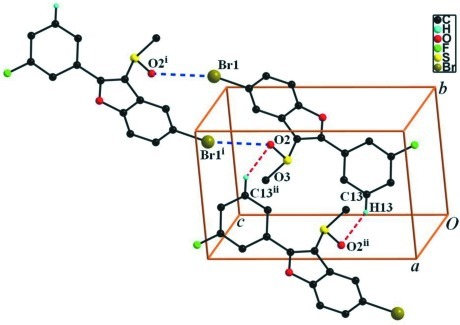
A view of the C—H···O and Br···O interactions (dotted lines) in the crystal structure of the title compound. H atoms non-participating in hydrogen-bonding were omitted for clarity. [Symmetry codes: (i) - x + 1, - y + 2, - z + 2 ; (ii) - x + 2, - y + 1, - z + 1.]

## supplementary crystallographic information

### Comment

As a part of our ongoing study of 5-bromo-3-methylsulfinyl-1-benzofuran derivatives containing 2-phenyl (Choi *et al.*, 2007) and 2-(4-fluorophenyl) (Choi *et al.*, 2010) substituents, we report herein the crystal structure of the title compound.

In the title molecule (Fig. 1), the benzofuran unit is essentially planar, with a mean deviation of 0.014 (1) Å from the least-squares plane defined by the nine constituent atoms. The dihedral angle between the 3-fluorophenyl ring and the mean plane of the benzofuran fragment is 30.77 (6)°. In the crystal structure (Fig. 2), molecules are connected by weak intermolecular C—H···O hydrogen bonds (Table 1), and by Br···O halogen-bondings between the bromine atom and the O atom of the S═O unit [Br1···O2^i^ = 3.214 (1) Å, C4—Br1···O2^i^ = 163.28 (5)°] (Politzer *et al.*, 2007).

### Experimental

3-Chloroperoxybenzoic acid (77%, 202 mg, 0.9 mmol) was added in small portions to a stirred solution of 5-bromo-2-(3-fluorophenyl)-3-methylsulfanyl-1-benzofuran (270 mg, 0.9 mmol) in dichloromethane (30 mL) at 273 K. After being stirred at room temperature for 5h, the mixture was washed with saturated sodium bicarbonate solution and the organic layer was separated, dried over magnesium sulfate, filtered and concentrated at reduced pressure. The residue was purified by column chromatography (hexane–ethyl acetate, 1:1 v/v) to afford the title compound as a colorless solid [yield 73%, m.p. 447–448 K; *R*_f_ = 0.55 (hexane–ethyl acetate, 1:1 v/v)]. Single crystals suitable for X-ray diffraction were prepared by slow evaporation of a solution of the title compound in acetone at room temperature.

### Refinement

All H atoms were positioned geometrically (C—H = 0.95 Å for the aryl and 0.98 Å for the methyl H atoms) and refined using a riding model, with *U*_iso_(H) = 1.2*U*_eq_(C) for aryl and 1.5*U*_eq_(C) for methyl H atoms. The positions of methyl H atoms were optimized rotationally.

### Crystal data

**Table:**

C_15_H_10_BrFO_2_S	*Z* = 2
*M**_r_* = 353.20	*F*(000) = 352
Triclinic, *P*1	*D*_x_ = 1.756 Mg m^−^^3^
Hall symbol: -P 1	Mo *K*α radiation, λ = 0.71073 Å
*a* = 8.0494 (1) Å	Cell parameters from 7435 reflections
*b* = 8.5317 (1) Å	θ = 2.6–27.5°
*c* = 10.7110 (2) Å	µ = 3.24 mm^−^^1^
α = 87.624 (1)°	*T* = 173 K
β = 81.378 (1)°	Block, colourless
γ = 66.709 (1)°	0.36 × 0.29 × 0.25 mm
*V* = 667.87 (2) Å^3^	

### Data collection

**Table:**

Bruker SMART APEXII CCD diffractometer	3059 independent reflections
Radiation source: rotating anode	2835 reflections with *I* > 2σ(*I*)
Graphite multilayer monochromator	*R*_int_ = 0.038
Detector resolution: 10.0 pixels mm^-1^	θ_max_ = 27.5°, θ_min_ = 1.9°
φ and ω scans	*h* = −10→10
Absorption correction: multi-scan (*SADABS*; Bruker, 2009)	*k* = −11→11
*T*_min_ = 0.391, *T*_max_ = 0.501	*l* = −13→13
11667 measured reflections	

### Refinement

**Table:**

Refinement on *F*^2^	Primary atom site location: structure-invariant direct methods
Least-squares matrix: full	Secondary atom site location: difference Fourier map
*R*[*F*^2^ > 2σ(*F*^2^)] = 0.025	Hydrogen site location: difference Fourier map
*wR*(*F*^2^) = 0.064	H-atom parameters constrained
*S* = 1.07	*w* = 1/[σ^2^(*F*_o_^2^) + (0.0304*P*)^2^ + 0.2245*P*] where *P* = (*F*_o_^2^ + 2*F*_c_^2^)/3
3059 reflections	(Δ/σ)_max_ = 0.003
182 parameters	Δρ_max_ = 0.36 e Å^−^^3^
0 restraints	Δρ_min_ = −0.46 e Å^−^^3^

### Special details

**Table:**

Geometry. All esds (except the esd in the dihedral angle between two l.s. planes) are estimated using the full covariance matrix. The cell esds are taken into account individually in the estimation of esds in distances, angles and torsion angles; correlations between esds in cell parameters are only used when they are defined by crystal symmetry. An approximate (isotropic) treatment of cell esds is used for estimating esds involving l.s. planes.
Refinement. Refinement of F^2^ against ALL reflections. The weighted R-factor wR and goodness of fit S are based on F^2^, conventional R-factors R are based on F, with F set to zero for negative F^2^. The threshold expression of F^2^ > 2sigma(F^2^) is used only for calculating R-factors(gt) etc. and is not relevant to the choice of reflections for refinement. R-factors based on F^2^ are statistically about twice as large as those based on F, and R- factors based on ALL data will be even larger.

### Fractional atomic coordinates and isotropic or equivalent isotropic displacement parameters (Å^2^)

**Table:**

	*x*	*y*	*z*	*U*_iso_*/*U*_eq_	
Br1	0.17500 (3)	1.14433 (2)	1.025572 (16)	0.03170 (8)	
S1	0.78664 (6)	0.69925 (5)	0.60864 (4)	0.02294 (10)	
F1	0.46013 (17)	0.70426 (15)	0.04748 (10)	0.0391 (3)	
O1	0.29411 (16)	0.86282 (14)	0.50431 (11)	0.0227 (2)	
O2	0.81884 (18)	0.83090 (16)	0.67671 (13)	0.0309 (3)	
C1	0.5524 (2)	0.78361 (19)	0.59217 (15)	0.0207 (3)	
C2	0.4029 (2)	0.89580 (19)	0.68141 (15)	0.0205 (3)	
C3	0.3839 (2)	0.9576 (2)	0.80375 (15)	0.0224 (3)	
H3	0.4859	0.9297	0.8475	0.027*	
C4	0.2095 (2)	1.0613 (2)	0.85784 (16)	0.0236 (3)	
C5	0.0559 (2)	1.1060 (2)	0.79601 (17)	0.0260 (4)	
H5	−0.0611	1.1781	0.8375	0.031*	
C6	0.0740 (2)	1.0455 (2)	0.67483 (17)	0.0254 (4)	
H6	−0.0279	1.0741	0.6308	0.030*	
C7	0.2488 (2)	0.9411 (2)	0.62143 (15)	0.0213 (3)	
C8	0.4801 (2)	0.7684 (2)	0.48847 (15)	0.0209 (3)	
C9	0.5542 (2)	0.6726 (2)	0.36924 (16)	0.0214 (3)	
C10	0.4711 (2)	0.7376 (2)	0.26146 (16)	0.0235 (3)	
H10	0.3691	0.8438	0.2646	0.028*	
C11	0.5423 (3)	0.6428 (2)	0.15148 (16)	0.0264 (4)	
C12	0.6896 (3)	0.4891 (2)	0.14076 (17)	0.0288 (4)	
H12	0.7343	0.4279	0.0625	0.035*	
C13	0.7716 (3)	0.4254 (2)	0.24751 (17)	0.0279 (4)	
H13	0.8746	0.3197	0.2425	0.033*	
C14	0.7036 (2)	0.5154 (2)	0.36110 (17)	0.0251 (4)	
H14	0.7589	0.4701	0.4341	0.030*	
C15	0.7821 (3)	0.5407 (2)	0.7231 (2)	0.0362 (5)	
H15A	0.9036	0.4827	0.7483	0.054*	
H15B	0.7470	0.4572	0.6862	0.054*	
H15C	0.6931	0.5958	0.7974	0.054*	

### Atomic displacement parameters (Å^2^)

**Table:**

	*U*^11^	*U*^22^	*U*^33^	*U*^12^	*U*^13^	*U*^23^
Br1	0.02857 (12)	0.03822 (11)	0.02513 (11)	−0.01096 (8)	0.00206 (7)	−0.00900 (7)
S1	0.0157 (2)	0.02387 (19)	0.0279 (2)	−0.00585 (15)	−0.00481 (16)	0.00069 (15)
F1	0.0440 (7)	0.0454 (6)	0.0252 (6)	−0.0113 (5)	−0.0152 (5)	−0.0005 (5)
O1	0.0172 (6)	0.0260 (6)	0.0224 (6)	−0.0047 (5)	−0.0053 (4)	−0.0020 (4)
O2	0.0266 (7)	0.0292 (6)	0.0416 (8)	−0.0129 (5)	−0.0134 (6)	0.0013 (5)
C1	0.0163 (8)	0.0212 (7)	0.0238 (8)	−0.0065 (6)	−0.0034 (6)	0.0000 (6)
C2	0.0172 (8)	0.0205 (7)	0.0234 (8)	−0.0069 (6)	−0.0032 (6)	0.0009 (6)
C3	0.0192 (8)	0.0248 (8)	0.0233 (8)	−0.0081 (6)	−0.0050 (6)	0.0009 (6)
C4	0.0241 (9)	0.0245 (7)	0.0222 (8)	−0.0102 (7)	−0.0013 (7)	−0.0016 (6)
C5	0.0185 (8)	0.0243 (8)	0.0309 (9)	−0.0047 (6)	−0.0003 (7)	−0.0012 (7)
C6	0.0167 (8)	0.0264 (8)	0.0303 (9)	−0.0047 (6)	−0.0058 (7)	−0.0002 (7)
C7	0.0199 (8)	0.0213 (7)	0.0223 (8)	−0.0071 (6)	−0.0043 (6)	0.0004 (6)
C8	0.0162 (8)	0.0209 (7)	0.0237 (8)	−0.0053 (6)	−0.0035 (6)	0.0009 (6)
C9	0.0204 (8)	0.0237 (7)	0.0224 (8)	−0.0107 (6)	−0.0035 (6)	−0.0016 (6)
C10	0.0206 (8)	0.0244 (7)	0.0271 (9)	−0.0096 (6)	−0.0059 (7)	0.0006 (6)
C11	0.0278 (10)	0.0327 (9)	0.0227 (8)	−0.0147 (7)	−0.0084 (7)	0.0022 (7)
C12	0.0287 (10)	0.0329 (9)	0.0259 (9)	−0.0139 (8)	0.0000 (7)	−0.0065 (7)
C13	0.0234 (9)	0.0256 (8)	0.0319 (9)	−0.0069 (7)	−0.0018 (7)	−0.0046 (7)
C14	0.0234 (9)	0.0250 (8)	0.0264 (8)	−0.0077 (7)	−0.0071 (7)	0.0007 (6)
C15	0.0331 (11)	0.0301 (9)	0.0502 (12)	−0.0143 (8)	−0.0191 (9)	0.0156 (8)

### Geometric parameters (Å, º)

**Table:**

Br1—C4	1.8994 (17)	C6—C7	1.382 (2)
S1—O2	1.4884 (13)	C6—H6	0.9500
S1—C1	1.7662 (17)	C8—C9	1.459 (2)
S1—C15	1.7951 (19)	C9—C14	1.398 (2)
F1—C11	1.3604 (19)	C9—C10	1.405 (2)
O1—C7	1.376 (2)	C10—C11	1.371 (2)
O1—C8	1.379 (2)	C10—H10	0.9500
C1—C8	1.362 (2)	C11—C12	1.372 (3)
C1—C2	1.449 (2)	C12—C13	1.389 (3)
C2—C7	1.393 (2)	C12—H12	0.9500
C2—C3	1.397 (2)	C13—C14	1.384 (2)
C3—C4	1.381 (2)	C13—H13	0.9500
C3—H3	0.9500	C14—H14	0.9500
C4—C5	1.402 (2)	C15—H15A	0.9800
C5—C6	1.382 (2)	C15—H15B	0.9800
C5—H5	0.9500	C15—H15C	0.9800
			
Br1···O2^i^	3.2137 (13)		
			
C4—Br1—O2^i^	163.28 (5)	C1—C8—C9	134.47 (15)
O2—S1—C1	107.07 (7)	O1—C8—C9	114.71 (14)
O2—S1—C15	105.85 (9)	C14—C9—C10	119.48 (15)
C1—S1—C15	97.64 (9)	C14—C9—C8	121.18 (15)
C7—O1—C8	106.58 (12)	C10—C9—C8	119.32 (15)
C8—C1—C2	106.97 (14)	C11—C10—C9	117.81 (16)
C8—C1—S1	126.42 (13)	C11—C10—H10	121.1
C2—C1—S1	126.36 (12)	C9—C10—H10	121.1
C7—C2—C3	119.31 (15)	F1—C11—C10	117.89 (16)
C7—C2—C1	105.05 (14)	F1—C11—C12	118.26 (16)
C3—C2—C1	135.62 (15)	C10—C11—C12	123.84 (16)
C4—C3—C2	116.65 (15)	C11—C12—C13	118.18 (16)
C4—C3—H3	121.7	C11—C12—H12	120.9
C2—C3—H3	121.7	C13—C12—H12	120.9
C3—C4—C5	123.30 (16)	C14—C13—C12	120.14 (16)
C3—C4—Br1	118.41 (13)	C14—C13—H13	119.9
C5—C4—Br1	118.28 (13)	C12—C13—H13	119.9
C6—C5—C4	120.26 (16)	C13—C14—C9	120.54 (16)
C6—C5—H5	119.9	C13—C14—H14	119.7
C4—C5—H5	119.9	C9—C14—H14	119.7
C7—C6—C5	116.17 (16)	S1—C15—H15A	109.5
C7—C6—H6	121.9	S1—C15—H15B	109.5
C5—C6—H6	121.9	H15A—C15—H15B	109.5
O1—C7—C6	125.06 (15)	S1—C15—H15C	109.5
O1—C7—C2	110.60 (14)	H15A—C15—H15C	109.5
C6—C7—C2	124.30 (16)	H15B—C15—H15C	109.5
C1—C8—O1	110.79 (14)		
			
O2—S1—C1—C8	139.58 (15)	C1—C2—C7—C6	178.75 (16)
C15—S1—C1—C8	−111.19 (16)	C2—C1—C8—O1	−0.06 (18)
O2—S1—C1—C2	−33.90 (16)	S1—C1—C8—O1	−174.57 (12)
C15—S1—C1—C2	75.34 (16)	C2—C1—C8—C9	−177.94 (18)
C8—C1—C2—C7	−0.52 (18)	S1—C1—C8—C9	7.5 (3)
S1—C1—C2—C7	174.00 (13)	C7—O1—C8—C1	0.63 (18)
C8—C1—C2—C3	177.77 (18)	C7—O1—C8—C9	178.96 (14)
S1—C1—C2—C3	−7.7 (3)	C1—C8—C9—C14	30.3 (3)
C7—C2—C3—C4	0.2 (2)	O1—C8—C9—C14	−147.50 (16)
C1—C2—C3—C4	−177.90 (18)	C1—C8—C9—C10	−151.18 (19)
C2—C3—C4—C5	−0.4 (3)	O1—C8—C9—C10	31.0 (2)
C2—C3—C4—Br1	178.50 (12)	C14—C9—C10—C11	−0.3 (3)
C3—C4—C5—C6	0.2 (3)	C8—C9—C10—C11	−178.86 (15)
Br1—C4—C5—C6	−178.66 (13)	C9—C10—C11—F1	178.95 (15)
C4—C5—C6—C7	0.1 (3)	C9—C10—C11—C12	−0.2 (3)
C8—O1—C7—C6	−178.78 (16)	F1—C11—C12—C13	−179.08 (16)
C8—O1—C7—C2	−0.97 (18)	C10—C11—C12—C13	0.1 (3)
C5—C6—C7—O1	177.22 (15)	C11—C12—C13—C14	0.6 (3)
C5—C6—C7—C2	−0.3 (3)	C12—C13—C14—C9	−1.2 (3)
C3—C2—C7—O1	−177.70 (14)	C10—C9—C14—C13	1.0 (3)
C1—C2—C7—O1	0.92 (18)	C8—C9—C14—C13	179.54 (17)
C3—C2—C7—C6	0.1 (3)		

Symmetry code: (i) −*x*+1, −*y*+2, −*z*+2.

### Hydrogen-bond geometry (Å, º)

**Table:**

*D*—H···*A*	*D*—H	H···*A*	*D*···*A*	*D*—H···*A*
C13—H13···O2^ii^	0.95	2.55	3.356 (2)	142

Symmetry code: (ii) −*x*+2, −*y*+1, −*z*+1.
